# Amyloid biomarkers as predictors of conversion from mild cognitive impairment to Alzheimer’s dementia: a comparison of methods

**DOI:** 10.1186/s13195-020-00721-3

**Published:** 2020-11-19

**Authors:** Arnd Sörensen, Ganna Blazhenets, Florian Schiller, Philipp Tobias Meyer, Lars Frings, for the Alzheimer Disease Neuroimaging Initiative

**Affiliations:** Department of Nuclear Medicine, Medical Center - University of Freiburg, Faculty of Medicine, University of Freiburg, Hugstetter Str. 55, 79106 Freiburg, Germany

**Keywords:** Amyloid biomarkers, Mild cognitive impairment, Alzheimer’s dementia, Conversion prediction, PET image evaluation

## Abstract

**Background:**

Amyloid-β (Aβ) PET is an established predictor of conversion from mild cognitive impairment (MCI) to Alzheimer’s dementia (AD). We compared three PET (including an approach based on voxel-wise Cox regression) and one cerebrospinal fluid (CSF) outcome measures in their predictive power.

**Methods:**

Datasets were retrieved from the ADNI database. In a training dataset (*N* = 159), voxel-wise Cox regression and principal component analyses were used to identify conversion-related regions (Cox-VOI and AD conversion-related pattern (ADCRP), respectively). In a test dataset (*N* = 129), the predictive value of mean normalized ^18^F-florbetapir uptake (SUVR) in AD-typical brain regions (composite SUVR) or the Cox-VOI and the pattern expression score (PES) of ADCRP and CSF Aβ_42_/Aβ_40_ as predictors were compared by Cox models (corrected for age and sex).

**Results:**

All four Aβ measures were significant predictors (*p* < 0.001). Prediction accuracies (Harrell’s *c*) showed step-wise significant increases from Cox-SUVR (*c* = 0.71; HR = 1.84 per *Z*-score increase), composite SUVR (*c* = 0.73; HR = 2.18), CSF Aβ_42_/Aβ_40_ (*c* = 0.75; HR = 3.89) to PES (*c* = 0.77; HR = 2.71).

**Conclusion:**

The PES of ADCRP is the most predictive Aβ PET outcome measure, comparable to CSF Aβ_42_/Aβ_40_, with a slight but statistically significant advantage.

## Introduction

Amyloid-β (Aβ) PET (e.g., using ^18^F-florbetapir, also known as ^18^F-AV-45) is an established biomarker for Aβ pathology [1] and might hence be used to predict conversion from mild cognitive impairment (MCI) to Alzheimer’s dementia (AD) [2–5]. Earlier studies commonly used binary Aβ outcome measures based on visual reads or volume of interest (VOI) analyses [2–4] or continuous Aβ measures relying on composite anatomical regions (i.e., treating all voxels equally) [5]. However, we recently demonstrated that voxel-wise principal component analysis (PCA) provides an AD-specific covariance pattern among voxels, which is superior to the aforementioned conventional approaches [6]. Another promising prognostic method in PET data analysis is voxel-wise Cox regression, which we recently applied to ^18^F-FDG PET in MCI [7]. However, the best image evaluation method is still a matter of debate.

Aside from Aβ PET, the Aβ concentration in cerebrospinal fluid (CSF) is also an established non-imaging biomarker for Aβ pathology [8]. The ratio of the concentration of Aβ_42_ to the concentration of Aβ_40_ (Aβ_42_/Aβ_40_) showed the best diagnostic performance among different evaluation methods [9, 10].

While many studies consider CSF Aβ and Aβ PET to be equally capable of predicting cognitive decline [11–16], some find a slight advantage for Aβ PET [17] in this regard. On the other hand, recent findings suggest that CSF indicates abnormal Aβ accumulation before Aβ PET in the earliest stages of the disease [18] and that Aβ PET is more strongly connected to disease progression [19].

Against this background, we used a large dataset from the Alzheimer’s disease neuroimaging initiative (ADNI) to compare the aforementioned three continuous Aβ PET outcome measures and the Aβ_42_/Aβ_40_ ratio in CSF in their ability to predict conversion from MCI to AD. We used a training dataset, to which voxel-wise Cox regression and PCA were applied to identify conversion-related regions, and a test dataset, by use of which all four methods were prospectively compared.

## Material and methods

### Subjects

All data used in the present study was provided by the ADNI database (ClinicalTrials.gov Identifier: NCT00106899), and comprehensive information about the ADNI project can be found at the official website (www.adni-info.org).

For our previous study [6], 319 ^18^F-AV-45 PET scans were retrieved from the ADNI database. Patients with the following criteria were included: MCI diagnosis (“DX-Score” 2, suspected incipient Alzheimer disease with subjective and objective memory deficits) and a baseline ^18^F-AV-45 scan, at least 25 points on Mini-Mental State Examination (MMSE), follow-up time of at least 6 months, and no bidirectional change of diagnosis (MCI to AD and back). This dataset was randomly split into two equally sized cohorts: a training and a test dataset. The training dataset has been used for voxel-wise Cox regression and PCA to identity the Cox-VOI in the present study (see below) and the ADCRP in our earlier study [6]. The test dataset for the present study was further reduced to a subset of 129 patients with available data on amyloid-β concentration in the CSF. Details on clinical and demographic characteristics can be found in Table 1.

**Table 1:** Clinical and demographic characteristics of the included Alzheimer’s Disease Neuroimaging Initiative (ADNI) participants

	Training dataset (***n*** = 159)		Test dataset (***n*** = 129)	
	MCI-c (*n* = 41)	MCI-nc (*n* = 118)	MCI-c (*n* = 29)	MCI-nc (*n* = 101)
Mean age (± S.D.) [years]	72 ± 7	73 ± 8	73 ± 7	73 ± 8
Sex [m/f]	14/27	55/63	15/14	59/42
Mean Aβ_42_/Aβ_40_ in CSF (± S.D.)	Not assessed		0.10 ± 0.06	0.15 ± 0.06
PES of ADCRP (± S.D.)	18 ± 19	− 6 ± 18	16 ± 13	− 2 ± 17
Median follow-up time (95% C.I.) [months]	48 (36–51)		47 (35–51)	
Cox-SUVR (± S.D.)	1.7 ± 0.3	1.4 ± 0.2	1.6 ± 0.2	1.5 ± 0.2
Composite SUVR (± S.D.)	Not assessed		1.6 ± 0.2	1.4 ± 0.2

*MCI-c* mild cognitive impairment–converters, *MCI-nc* MCI non-converters, *S.D.* standard deviation, *CSF* cerebrospinal fluid, *PES* pattern expression score, *SUVR* standardized uptake value ratio

### ^18^F-AV-45 PET data and image preprocessing

Four 5-min frames were used (50 to 70 min after injection of ^18^F-AV-45). A motion correction was applied if necessary. All frames were summed into a single image dataset. After spatial normalization to an in-house template (^18^F-Florbetapir, constructed from nine Aβ-positive and seven Aβ-negative elderly normal controls) in MNI space, spatial smoothing with a 12-mm FWHM isotropic Gaussian kernel was applied. Full details on PET acquisition protocols can be found on the ADNI website.

### PET image analysis: training dataset

For each voxel, independently, a Cox model was fitted with the *z*-scaled SUVR as a predictor variable, adjusted for age and sex. Among those voxels that showed a significant association between SUVR and conversion from MCI to AD (FDR-corrected, *p* < 0.01), the 20% of voxels with the highest hazard ratios (HR) were combined into the “Cox-VOI.”

The same training dataset was used in our previous study [6] to identify the AD conversion-related pattern (ADCRP), which was also used in the present study for the evaluation of the test dataset.

### Cox regressions: test dataset

In the test dataset, four Cox models were built in order to compare their prognostic performance concerning MCI-to-AD conversion. Each model included one of the four Aβ measures as the main predictor variable (all *z*-scaled), as well as age and sex as covariates:

1. *Composite SUVR*: the mean standardized uptake value ratio (SUVR, reference region: cerebellum) was calculated within a VOI comprising anatomical regions with the highest Aβ load in AD (established in a previous study [20] using Pittsburgh compound B).
2. *Cox-SUVR*: the mean SUVR within the Cox-VOI was read-out and weighted in a voxel-wise fashion by its HR (calculated in the training dataset; thus, voxel with a higher predictive value contributed more).
3. *PES of ADCRP*: the individual pattern expression score (PES) was calculated for the ADCRP that has been established in our previous study [6]. The PES was evaluated by the topographic profile rating algorithm, as described in [21].
4. CSF Aβ_42_/Aβ_40_: the Aβ_42_/Aβ_40_ concentration ratio from CSF was used the main predictor variable.

## Results

### Training dataset

**Fig. 1:**
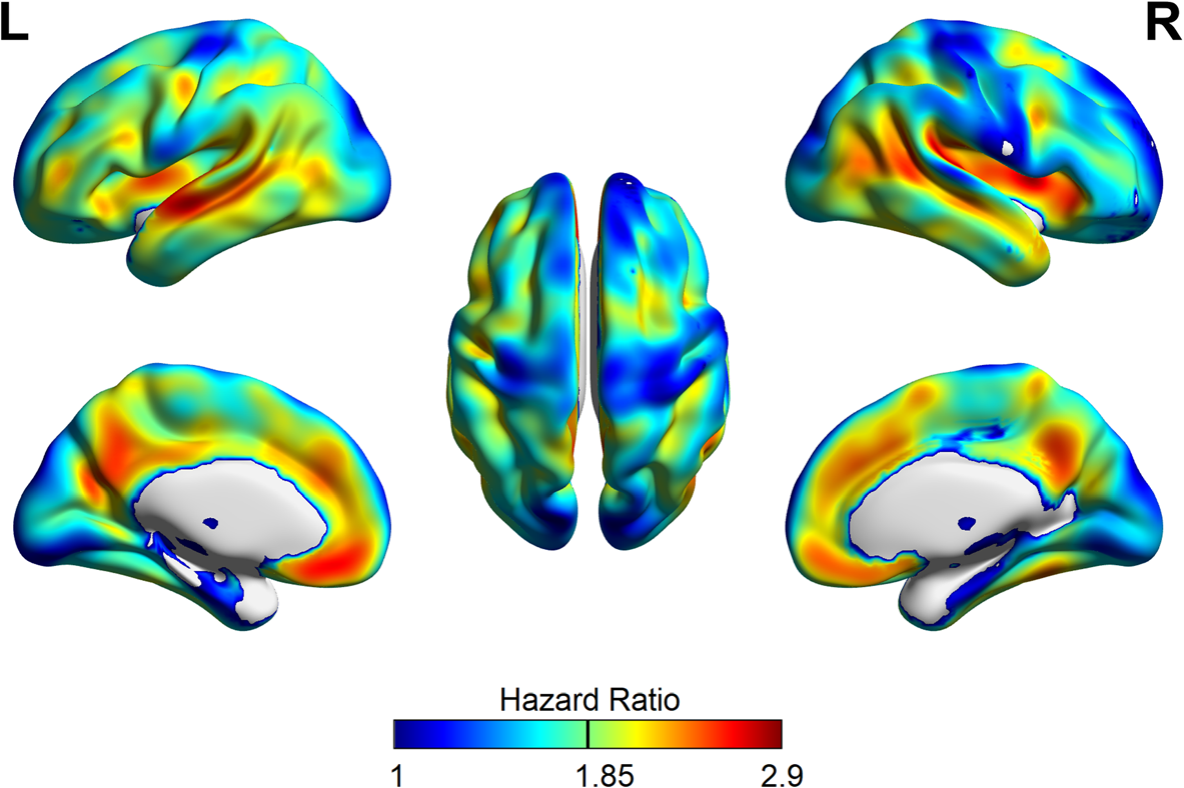
Surface projections of the hazard ratios (HRs) from the voxel-wise Cox regressions in the training dataset. HR is expressed per one unit increase of the *z*-scaled standardized uptake value ratio (SUVR; reference region: cerebellum) of ^18^F-florbetapir

**Fig. 2:**
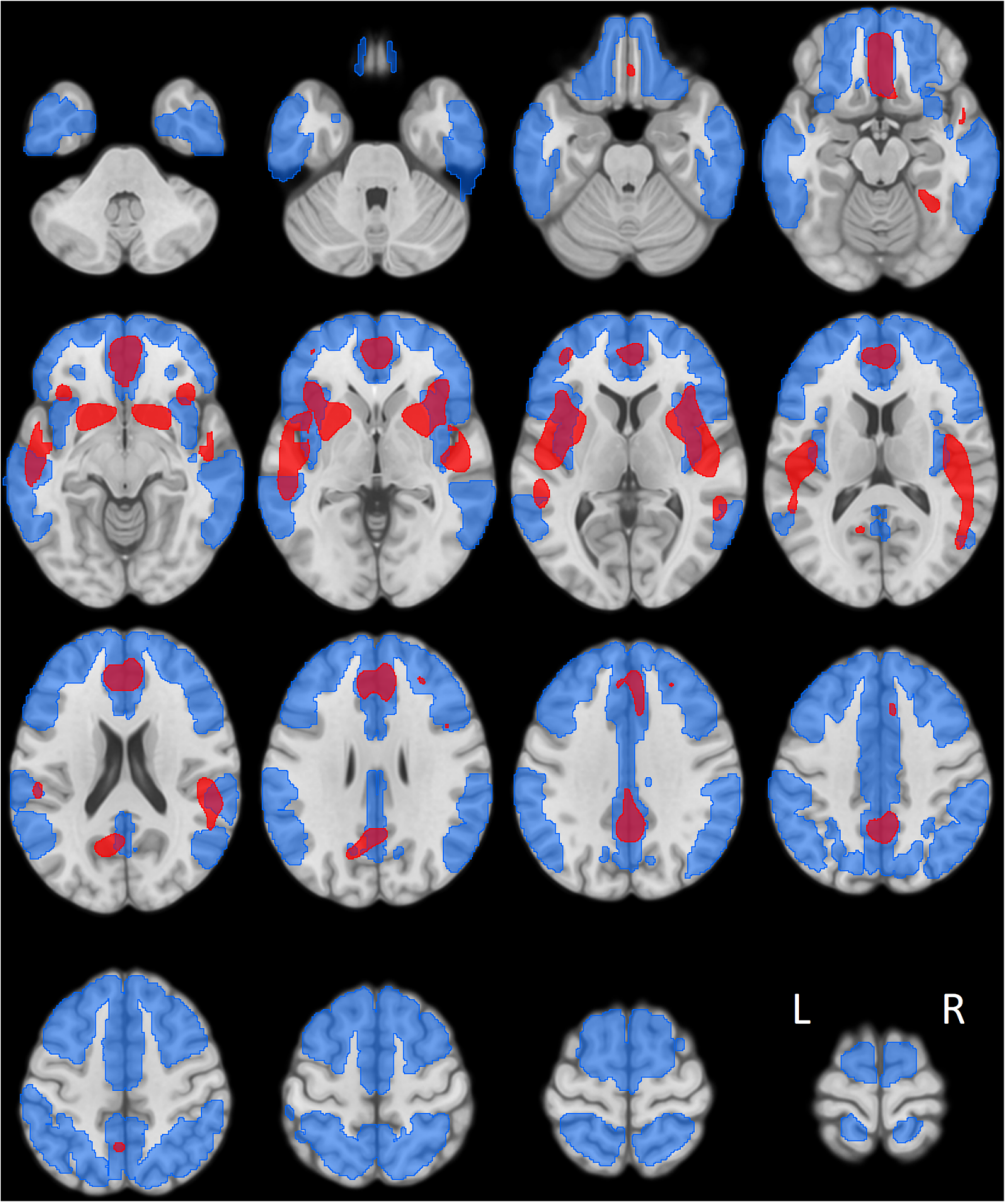
Volume of interest (VOI) overlays (onto the MNI-152 MRI template) showing significant voxels with top 20% hazard ratios (HRs) from voxel-wise Cox regressions in the training dataset (red, Cox-VOI) used to assess Cox-SUVR and the anatomical VOI (blue; taken from [20]) employed for composite SUVR calculation

Figure 1 depicts three-dimensional surface projections of HRs calculated by voxel-wise Cox regression in the training dataset, which follows the known distribution of Aβ pathology in AD. Voxels with the top 20% of HR are illustrated in Fig. 2 (red regions, 79 ml), which cover parts of the striatum and mesial frontal and superior temporal cortices as well as the precuneus and insula. These regions only partially overlap (42 ml) with the larger anatomical VOI used to calculate the composite SUVR (Fig. 2, blue regions, 584 ml).

### Test dataset

**Fig. 3:**
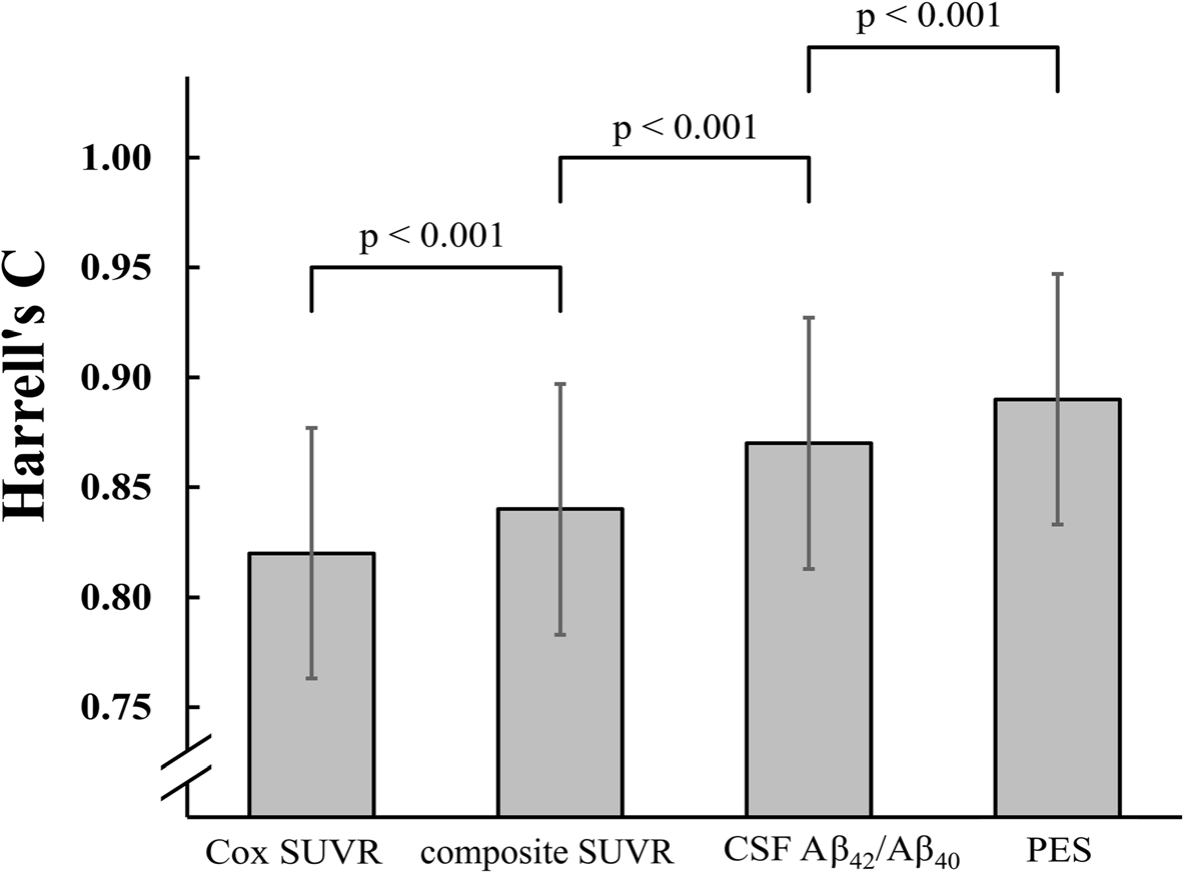
Prediction concordance (Harrell’s *c*) and respective confidence intervals of the four Cox regression models (corrected for age and sex). *P* values were derived from pair-wise likelihood ratio tests

All four Cox models significantly predicted MCI-to-AD conversion in the test dataset (all Wald tests *p* < 0.001). Pairwise comparisons between models (Fig. 3), using the likelihood ratio test, revealed significant step-wise improvements (*p* < 0.001) from the model with Cox-SUVR (HR = 1.84 per *Z*-score increase [95% C.I. 1.31–2.56]) with a concordance of Harrell’s *c* = 0.71 (95% C.I. 0.59–0.82) to the model incorporating composite SUVR (HR = 2.18 [1.51–3.16]) with *c* = 0.73 (0.62–0.84), to the model relying on CSF Aβ_42_/Aβ_40_ (HR = 3.89 [2.10–7.19]) with *c* = 0.75 (0.65–0.87), and, finally, to the model using PES of ADCRP as a predictor (HR of 2.71 [1.78–4.13]) with *c* = 0.77 (0.66–0.89).

## Discussion

In the present study, all three tested Aβ PET outcome measures and the CSF Aβ_42_/Aβ_40_ ratio were able to predict the development of AD in patients diagnosed with MCI. Among these Cox models, each containing the main predictor variable and all corrected for age and sex, the model with the PES of ADCRP showed the highest concordance (Harrell’s *c*). Binary outcome measures such as visual reads or threshold-based methods have been neglected for this study, as we have shown in our previous work [6] that binary measures perform worse than continuous Aβ PET outcome measures.

Cox-SUVR represents a novel Aβ PET outcome measure, which we explored based on our previous observation that voxel-wise Cox regression is a promising predictor of MCI-to-AD conversion when applied to FDG PET data [7]. In the training dataset, we tested several HR thresholds (using the top 50%, 30%, 20%, and 10%) for Cox-SUVR calculation. All threshold-based approaches showed improvement over using simply all significant voxels. The 20% threshold performed best and was thus chosen. The distribution and magnitude of voxel-wise HRs (Fig. 1) are largely in agreement with regions of known Aβ deposition in AD [22–25], which were used for the calculation of composite SUVR as an established prognostic marker [20]. However, the Cox-VOI (79 ml) was much smaller than the anatomical VOI (584 ml), with only little overlap (42 ml).

Both Aβ PET and determination of Aβ from CSF offer an excellent way to predict the development of Alzheimer’s disease: Aβ from CSF detects amyloid pathology earlier than Aβ PET [18], but both are predictors in their own right, such that patients with concordant CSF and amyloid PET findings have a worse prognosis than those with discordant findings [26, 27]. The choice regarding which of the two will be applied should depend on the availability and the patient’s preference.

Evaluation of Aβ PET by PES from PCA might easily be implemented to support clinical routine since Eidelberg [28] developed a freely available toolbox (Scanvp/SSMPCA toolbox available at the website of the Feinstein Institute for Medical Research, http://feinsteinneuroscience.org/software, [29]) for Statistical Parametric Mapping (SPM). The prognostic value of Aβ PET might also benefit from scanner development (higher spatial resolution and better signal-to-noise ratio). Finally, an ideal combination in the future of AD diagnosis might be that of Aβ PET with tau PET, allowing for a classification (regarding “A” and “T”) following the NIA-AA research framework.

## Limitations

In contrast to the novel Cox-SUVR, composite SUVR and the CSF Aβ_42_/Aβ_40_ ratio are established diagnostic and prognostic biomarkers of AD [1–5, 8]. Several other factors might be considered to contribute to the performance ranking obtained from our analyses: First, only for the derivation of the Cox-VOI the time-to-conversion information was used, but unexpectedly, it performed worst in the overall comparison. We assume, however, that time information might prove more beneficial in datasets with a larger inter-individual variability of time-to-conversion (the interquartile range was just 13 months in the test dataset). Secondly, while CSF Aβ_42_/Aβ_40_ is an integral measure for the production and clearance of Aβ at a given time and across all brain regions, Aβ PET represents a direct measurement of spatial Aβ accumulation. Thirdly, while all PET measures tested here provide regional weighting, this is only binary in the case of composite SUVR. By contrast, regional weighting is continuous in Cox-SUVR and PES. Last, the PES calculation includes not only regions with high Aβ accumulation or associated with an increased risk of conversion, but all voxels of the brain (i.e., possibly also patterns associated with lower risk or protective features). A combination of these factors might explain why the PES of ADCRP performed best in this comparison—by a small margin.

## Conclusion

All tested Aβ outcome measures significantly predicted conversion from MCI to AD. The PES of ADCRP is comparable to CSF Aβ_42_/Aβ_40_, with a slight but statistically significant advantage over CSF Aβ_42_/Aβ_40_.

## Data Availability

After registration, the initial imaging and patient metadata are available from the official ADNI website.

## Abbreviations

Aβ: Amyloid-β
Aβ_42_/Aβ_40_: Ratio of the concentration of Aβ_42_ to the concentration of Aβ_40_
AD: Alzheimer’s dementia
ADCRP: AD conversion-related pattern
ADNI: Alzheimer’s Disease Neuroimaging Initiative
CSF: Cerebrospinal fluid
^18^F-AV-45: ^18^F-florbetapir
FDR: False discovery rate
FWHM: Full width at half maximum
HR: Hazard ratio
MCI: Mild cognitive impairment
MMSE: Mini-Mental State Examination
MNI: Montreal Neurological Institute
PCA: Principal component analysis
PES: Pattern expression score
PET: Positron emission tomography
SPM: Statistical parametric mapping
SUVR: Standardized uptake value ratio
VOI: Volume of interest
